# Effects of thyroid-stimulating hormone and sensitivity to thyroid hormones on the risk of hyperuricemia in euthyroid adults

**DOI:** 10.20945/2359-4292-2024-0378

**Published:** 2025-09-24

**Authors:** Hongzhan Ding, Yanyu Liang, Yilin Wang, Kexin Zhang, Mengya Zhu, Yang Jing, Yong Xue, Xiaofang Chen, Hui Zhou, Chen Dong

**Affiliations:** 1 Department of Epidemiology and Statistics, School of Public Health, Medical College of Soochow University, Soochow, China; 2 Huai’an Third Hospital, Huai’an, China; 3 Suzhou Industrial Park Center for Disease Control and Prevention, Soochow, China

**Keywords:** Thyroid-stimulating hormone, thyroid hormone sensitivity, hyperuricemia, gout, Mendelian randomization

## Abstract

**Objective:**

The current study was conducted to investigate whether thyroid-stimulating hormone (TSH) and thyroid hormone sensitivity are associated with hyperuricemia probability in euthyroid population.

**Materials and methods:**

The observational analysis was based on a Chinese community-based cohort (n = 1,972). The prospective associations of TSH levels, TSH index (TSHI), thyrotrophic thyroxine resistance index (TT4RI), thyroid feedback quantile-based index (TFQI) and free triiodothyronine to free thyroxine (FT3/FT4) ratio with the risk of hyperuricemia were examined. Two-sample Mendelian randomization (MR) analysis was then used to test the causal effects of TSH on serum uric acid (SUA) levels and gout.

**Results:**

Among 1,972 participants with normal thyroid function, 244 new hyperuricemia cases were identified during follow-up. The results suggested that the higher levels of TSH (HR = 1.87, 95% CI: 1.28-2.73, p-value < 0.01), TSHI (HR = 2.02, 95% CI: 1.38-2.95, p-value < 0.01), TFQI (HR = 1.92, 95% CI: 1.33-2.76, p-value < 0.01) and TT4RI (HR = 1.93, 95% CI: 1.34-2.80, p-value < 0.01) were significantly associated with hyperuricemia incidence. The MR results further indicated causal effects of TSH on SUA levels (inverse variance weighting [IVW] β = 0.037, 95% CI: 0.017-0.057) and gout (IVW OR = 1.0018, 95% CI: 1.0004-1.0032).

**Conclusion:**

The higher levels of TSH, TSHI, TFQI and TT4RI are significantly associated with the risk of hyperuricemia in euthyroid population. The MR analysis supports the causal effects of TSH on SUA levels and gout.

## INTRODUCTION

Hyperuricemia, a major metabolic disorder, develops when uric acid levels rise above a threshold due to either excessive uric acid synthesis or insufficient uric acid excretion. Over the past decades, the increasing incidence of hyperuricemia has become a serious public health concern worldwide. In China, the prevalence of hyperuricemia increased from 11.1% to 14.0% between 2015 and 2019 (^1^). A number of previous epidemiologic studies have confirmed that hyperuricemia is a significant risk factor for several major chronic diseases, including gout, hypertension, cardiovascular disease, kidney disease, type 2 diabetes, and others (^2^-^5^). However, the pathogenesis of hyperuricemia is complex and not yet fully understood. Existing research has suggested several risk factors for the development of hyperuricemia, such as obesity, smoking, drinking, and dyslipidemia (^6^,^7^).

Thyroid-stimulating hormone (TSH) is produced and secreted by thyrotropic cells in the pituitary gland and is primarily responsible for regulating the activity of the thyroid gland. Under physiological conditions, the hypothalamic-pituitary-thyroid feedback loop maintains a constant level of TSH in the bloodstream. The pituitary gland is sensitive to small changes in serum thyroid hormone levels. When serum thyroid hormones levels fall below the set point, the pituitary gland releases TSH. The association between abnormal thyroid function and hyperuricemia and gout is certainly not new and has been discussed for several years. For example, results from a Chinese cross-sectional study reported that male participants with mild hypothyroidism had a 1.49-fold increased risk of hyperuricemia (^8^). In addition, See and cols. reported that hypothyroid and hyperthyroid status were associated with a 1.47-fold and 1.37-fold increased risk of gout, respectively (^9^). However, the issue of direct causality between TSH and hyperuricemia is still debated, especially in adults with normal thyroid function. In a Chinese cross-sectional study involving 19,013 participants, Yang and cols. suggested that men with elevated TSH levels might be at greater risk of hyperuricemia (^10^). However, the results of another Chinese cohort study did not reveal a significant association between TSH levels and the incidence of hyperuricemia in men and women (^11^).

The secretion of TSH is not only modulated by thyroid hormone levels but is also significantly influenced by pituitary and peripheral sensitivity to thyroid hormones. It has been reported that in cases of thyroid hormone resistance, elevated thyroid hormone levels may coexist with high TSH concentrations (^12^). Thyroid hormone resistance can be assessed by pituitary thyroid hormone sensitivity indices, such as the TSH index (TSHI), the thyrotrophic thyroxine resistance index (TT4RI), the thyroid feedback quantile-based index (TFQI), and the peripheral thyroid hormone sensitivity index, which is specifically calculated as the free triiodothyronine to free thyroxine (FT3/FT4) ratio (^12^-^14^). Elevated pituitary sensitivity indices in euthyroid individuals generally indicate pituitary resistance to thyroid hormones, meaning that even a slight decrease in thyroid hormone levels will trigger the secretion of TSH. In addition, pituitary resistance to thyroid hormones is usually accompanied by peripheral resistance to thyroid hormones, as manifested by decreased conversion efficiency of FT4 to FT3. This may represent a compensatory mechanism of the thyroid system and TSH secretion (^15^).

Using a Chinese community-based prospective cohort, the present study was conducted to assess the associations between TSH levels, thyroid hormone sensitivity indices (TSHI, TT4RI, TFQI, and FT3/FT4 ratio), and the risk of hyperuricemia in the euthyroid population. Furthermore, a two-sample Mendelian randomization (MR) analysis was conducted to investigate the potential association between genetic predisposition to TSH levels and serum uric acid (SUA) levels and gout, given that MR analysis can overcome limitations such as residual confounding and reverse causation seen in traditional observational studies (^16^).

## MATERIALS AND METHODS

### Cohort study

#### Study populations

**Figure S1:**
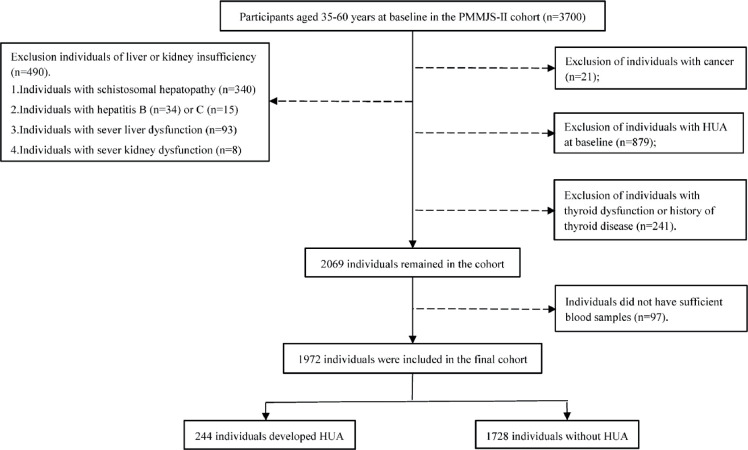
The workflow of the selection of participants from the PMMJS-II cohort.

This study was derived from “The Prevention of Metabolic Syndrome and Multi-metabolic Disorders in Jiangsu Province of China II (PMMJS-II)”, an ongoing community-based cohort study conducted in Soochow, China. Detailed baseline profiles of this cohort study have been reported before (^17^). Briefly, a total of 3,700 participants aged 35 to 60 years were recruited from June 2014 to May 2015. Follow-up surveys were carried out every two years thereafter until December 31, 2022. As shown in **Figure S1**, we excluded individuals with the following characteristics at baseline: hyperuricemia, severe liver or kidney insufficiency, cancer, thyroid dysfunction or history of thyroid disease, and insufficient blood samples. Eventually, 1,972 individuals were eligible for inclusion in the analysis. All participants provided written informed consent. The study protocol was approved by the Ethics Committee of Suzhou Industrial Park Center for Disease Control and Prevention (Soochow, China), and conducted in accordance with the ethical standards stated in the Declaration of Helsinki.

#### Data collection

Information on socio-demographics, lifestyle factors, health status, and medical history was collected from each participant using standard questionnaires. Standing height, body weight, waist circumference, and hip circumference were measured with participants wearing light indoor clothing and without shoes. Current smoking was defined as having smoked at least one cigarette a day for more than six months. Heavy drinking was defined as consuming alcohol > 40 g/day in males and > 30 g/day in females (^18^). Body mass index (BMI) was calculated as weight in kilograms divided by height in meters squared. Hypertension was defined as systolic blood pressure ≥ 140 mmHg or diastolic blood pressure ≥ 90 mmHg or self-reported diagnosis history of hypertension, or use of any anti-hypertensive medication.

Serum TSH, FT3, and FT4 levels were measured using electrochemiluminescence immunoassay (ECLIA) on an autoanalyzer MAGLUMI X8 (Snibe, China). The assay-specific reference ranges for TSH, FT3, and FT4 were 0.30-4.50 mIU/L, 3.08-6.47 pmol/L, and 11.45-22.14 pmol/L, respectively. Thyroid dysfunction was considered if the TSH, FT3 or FT4 were outside the reference range (^19^). Thyroid hormone sensitivity indices were calculated as follows: TFQI was calculated as the empirical cumulative distribution function cdf FT4-(1-cdf TSH), and the value of TFQI ranged from -1 to 1 (^12^). TT4RI was calculated as FT4 (pmol/L) × TSH (mIU/L) (^13^). TSHI was calculated as Ln TSH (mIU/L) + 0.1345 × FT4 (pmol/L) (^14^). The higher values of TFQI, TT4RI, and TSHI indices indicate lower pituitary sensitivity to thyroid hormone. Additionally, the FT3/FT4 ratio was calculated by dividing FT3 by FT4. A high FT3/FT4 ratio indicates higher peripheral sensitivity to thyroid hormones (^20^).

Biochemical tests, including SUA, creatinine, cholesterol (TC), triglycerides (TG), high-density lipoprotein cholesterol (HDL-C), low-density lipoprotein cholesterol (LDL-C), fasting plasma glucose (FPG), aspartate aminotransferase (AST), and alanine transaminase (ALT) were measured using an AU5800 analyzer (Beckman Coulter K.K.). Diabetes was defined as FPG ≥ 7.0 mmol/L, random glucose ≥ 11.1 mmol/L, self-reported diagnosis history of diabetes, or use of any glucose-lowering medication (^21^). Dyslipidemia was defined as TG ≥ 2.26 mmol/L and/or TC ≥ 6.22 mmol/L and/or LDL-C ≥ 4.14 mmol/L and/or HDL-C ≤ 1.04 mmol/L, or using of any lipid-lowering medication (^22^). The estimated glomerular filtration rate (eGFR) levels were calculated according to the 2021 Chronic Kidney Disease Epidemiology Collaboration (CKD-EPI) (^23^).

### Assessment of hyperuricemia

Hyperuricemia was defined based on any of the following criteria: SUA levels ≥ 420 µmol/L in males, or SUA levels ≥ 360 µmol/L in females, self-reported physician-diagnosed gout, taking anti-gout medication (^24^).

### Two-sample MR study

#### Data sources

Two-sample MR analyses were conducted using publicly released genome-wide association study (GWAS) summary statistics. The GWAS summary statistics for reference range TSH were obtained from the ThyroidOmics Consortium (^25^). The GWAS summary statistics for SUA levels and gout were collected from the United Kingdom Biobank datasets (^26^,^27^). Detailed information on the data sources contributing to our MR analyses is described in **Table S1**. We carefully selected summary statistics from the largest available GWAS meta-analyses and data with minimal sample overlap to ensure accurate and unbiased results. The MR analysis was based on summary-level data and thus required no ethical approval or informed consent.

#### Selection of genetic instruments

**Figure S2:**
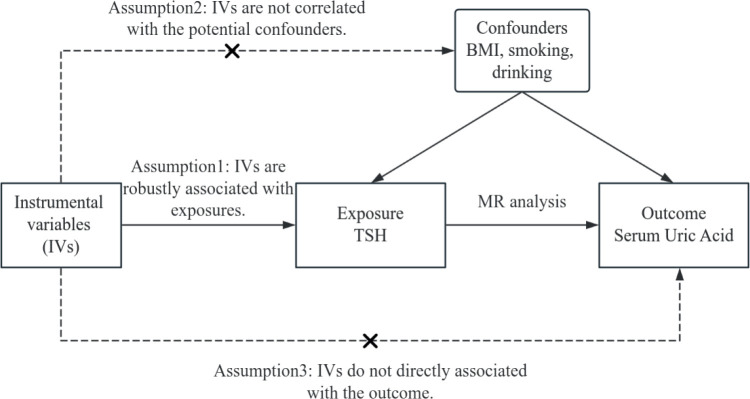
The flow diagram of Mendelian randomization analyses.

In the two-sample MR, we filtered instrumental variables (IVs) based on the three core MR assumptions (**Figure S2**). Assumption 1 is that IVs should be strongly associated with the exposure (p-value < 5 × 10^-8^). Assumption 2 is that IVs should not directly influence confounders between exposures and outcomes; therefore, Phenoscanner V2 was used to exclude significant single nucleotide polymorphisms (SNPs) that influence known confounders such as BMI, alcohol consumption, and smoking (^28^). Assumption 3 specifies that IVs should not directly affect outcomes other than via the exposures, so we excluded SNPs that might be outcome-related and used MR Egger regression to detect the bias caused by horizontal pleiotropy (^29^). All the selected SNPs were confirmed to be independently distributed without linkage disequilibrium (r^2^ < 0.001 within a distance of 10,000). Furthermore, the strength of each SNP was measured by F-statistics: R^2^/(1-R^2^) × [(N-K-1)/K], where R^2^ was the proportion of the exposure explained by the genetic variants, K was the number of included SNPs, and N was the sample size, to avoid weak-instrument bias (F > 10 suggested a low probability of weak-instrument bias) (^30^). The F-statistics of all the included SNPs were above the threshold of 10 (**Table S2**). Harmonization was performed to exclude palindromic and incompatible SNPs. The MR-PRESSO test was used to detect and exclude any outlier SNPs.

### Statistical analysis

For the cohort study, continuous variables are reported as mean ± standard deviation (SD) or median (interquartile range). Normality was tested using the Shapiro-Wilk test. Categorical variables are presented as cases (n) and percentages (%). Differences between the TSH quartiles (Q1-Q4) were tested using the chi-square test for categorical variables, the one-way ANOVA test for normally distributed variables, or the Kruskal-Wallis test for skewed distributions. Linear regression analyses were performed to assess the relationships between TSH, thyroid hormone sensitivity, and eGFR and SUA levels. Restricted cubic spline (RCS) models were also used to assess the dose-response associations, with knots placed at the 10th, 50th, and 90th percentiles. Cox proportional hazard models were used to calculate the hazard ratios (HRs) and 95% confidence intervals (CIs) for the associations between TSH or thyroid hormone sensitivity indices and hyperuricemia, adjusted for possible confounding factors such as age, sex, BMI, current smoking, heavy drinking, hypertension, diabetes, dyslipidemia, ALT, AST, and eGFR. Sensitivity analyses were performed to test the robustness of the results. Subgroup analyses were conducted according to age, sex, BMI, current smoking, heavy drinking, hypertension, diabetes, and dyslipidemia (^31^-^33^). The interactions between TSH levels and thyroid hormone sensitivity indices and the subgroup variables were assessed using the likelihood ratio test.

In the two-sample MR analysis, the inverse variance weighted (IVW) method was applied as the main MR analysis. Before that, Cochran’s Q test was performed, combined with *I^2^* statistics, to measure the heterogeneity across IVs. If there was strong evidence of heterogeneity, the random-effects IVW method was used as an alternative approach. To enhance the reliability of the causal inference, we also conducted several complementary analyses, including the weighted median, MR Egger, maximum likelihood, and robust adjusted profile score (MR RAPS) methods. This MR study was reported according to the STROBE-MR checklist (^34^).

Statistical analyses were performed using Statistical Analysis Software (SAS) version 9.4 (SAS Institute Inc., Cary, NC) and R version 4.3.2 (http://www.R-project.org). The RCS models were generated using the R package “rms”. In addition, the R package “mice” was used for multiple imputation of missing data. Two-sample MR analyses were performed using the “TwoSampleMR” package. p-values were two-tailed, and a p-value <0.05 was considered statistically significant.

## RESULTS

### Cohort study

**Table 1** describes the baseline characteristics of the participants. Among the 1,972 included participants, the median age was 50 (46, 55) years, and 64.1% were females. Individuals with lower TSH levels were more likely to be males (p-value < 0.01), with a higher proportion of smokers (p-value < 0.01) and heavy drinkers (p-value < 0.01), and had lower levels of TG (p-value = 0.03) and HDL-C (p-value = 0.02). As expected, serum FT4 and FT3 levels decreased with increasing TSH levels. After adjustment for age and sex, the FT3/FT4 ratio was negatively correlated with SUA levels. However, TSH, TFQI, TT4RI, and TSHI were not only negatively correlated with eGFR but also positively correlated with SUA levels (**Table S3**).

**Table 1:** Baseline characteristics of participants in the cohort study

Characteristics	TSH (mIU/L)				p-value
	Quartile 1	Quartile 2	Quartile 3	Quartile 4	
N	492	494	493	493	-
Age (years)	50 (46, 55)	49 (45, 53)	49 (46, 56)	50 (45, 56)	0.01
Male, n (%)	232 (47.2)	193 (39.1)	168 (34.1)	114 (23.1)	<0.01
Current smoking, n (%)	165 (33.5)	135 (27.3)	107 (21.7)	58 (11.8)	<0.01
Heavy drinking, n (%)	107 (21.7)	89 (18.0)	87 (17.6)	39 (7.9)	<0.01
Hypertension, n (%)	180 (36.6)	170 (34.4)	195 (39.6)	174 (35.3)	0.36
Diabetes, n (%)	51 (10.4)	43 (8.7)	54 (11.0)	40 (8.1)	0.38
Dyslipidemia, n (%)	173 (35.2)	170 (34.4)	163 (33.1)	148 (30.0)	0.34
BMI (kg/m^2^)	23.50±2.89	23.50±2.75	23.68±2.91	23.53±2.82	0.72
WHR	0.87±0.06	0.88±0.07	0.87±0.06	0.87±0.07	0.07
FT3 (pmol/L)	4.26 (3.94, 4.80)	4.29 (3.95, 4.80)	4.21 (3.90, 4.67)	4.19 (3.86, 4.58)	<0.01
FT4 (pmol/L)	12.85 (11.71, 13.98)	12.67 (11.57, 13.91)	12.48 (11.42, 13.68)	12.45 (11.45, 13.68)	<0.01
TSH (mIU/L)	0.87 (0.72, 0.98)	1.35 (1.23, 1.49)	2.00 (1.79, 2.26)	3.16 (2.87, 3.67)	<0.01
ALT (U/L)	22.07±15.04	21.46±14.79	22.36±17.37	20.41±13.13	0.19
AST (U/L)	23.24±8.79	22.81±8.93	24.17±11.16	23.13±7.20	0.11
TC (mmol/L)	4.79 (4.25, 5.34)	4.77 (4.21, 5.43)	4.79 (4.28, 5.37)	4.84 (4.25, 5.36)	0.88
TG (mmol/L)	1.06 (0.79, 1.48)	1.19 (0.82, 1.55)	1.21 (0.83, 1.63)	1.22 (0.86, 1.63)	0.03
LDL-C (mmol/L)	2.98 (2.55, 3.47)	2.98 (2.55, 3.52)	2.98 (2.55, 3.44)	2.98 (2.47, 3.43)	0.63
HDL-C (mmol/L)	1.18 (1.01, 1.35)	1.15 (1.00, 1.40)	1.18 (1.04, 1.38)	1.22 (1.07, 1.39)	0.02
eGFR(mL/min/1.73 m^2^)	103.8±9.49	104.95±8.93	103.78±8.57	103.97±9.62	0.14
SUA (µmol/L)	303 (245, 343)	296 (253, 342)	303 (264, 339)	292 (251, 333)	0.12
TFQI	-0.34±0.30	-0.12±0.30	0.11±0.31	0.35±0.29	<0.01
TT4RI	10.74 (8.96, 12.89)	16.98 (15.11, 19.41)	25.01 (21.70, 29.17)	39.87 (34.95, 46.51)	<0.01
TSHI	1.55±0.35	2.01±0.26	2.40±0.28	2.87±0.27	<0.01
FT3/FT4 ratio	0.34±0.06	0.35±0.06	0.35±0.06	0.34±0.05	0.48

Abbreviations: BMI, body mass index; WHR, waist-to-hip ratio; FT3, free triiodothyronine; FT4, free thyroxine; TSH, thyroid-stimulating hormone; ALT, alanine transaminase; AST, aspartate aminotransferase; TC, total cholesterol; TG, triglycerides; LDL-C, low-density lipoprotein cholesterol; HDL-C, high-density lipoprotein cholesterol; eGFR, estimated glomerular filtration rate; SUA, serum uric acid; TFQI, thyroid feedback quantile-based index; TT4RI, thyrotrophic thyroxine resistance index; TSHI, thyroid-stimulating hormone index.

**Figure 1:**
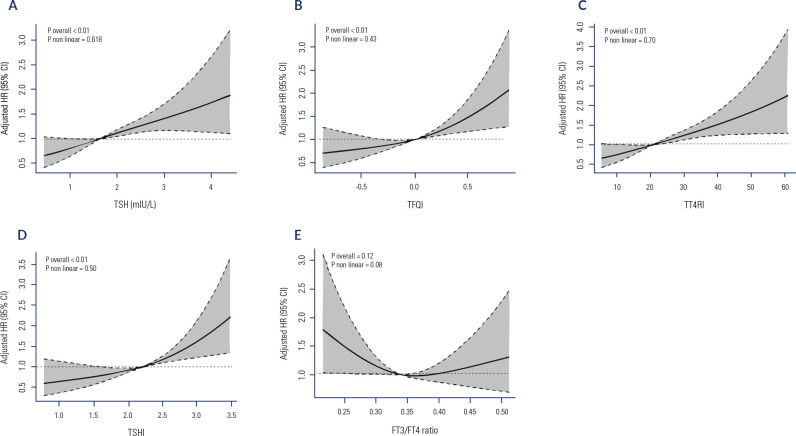
RCS analysis between TSH, TFQI, TT4RI, TSHI and FT3/FT4 ratio and the risk of hyperuricemia.

During 8.6 years of follow-up, 244 cases of hyperuricemia were identified. The dose-response relationships between TSH, thyroid hormone sensitivity indices, and hyperuricemia risk are shown in **Figure 1**. After adjusting for potential confounding factors, the results of the RCS analysis indicated that the risk of hyperuricemia increased with TSH, TFQI, TT4RI, and TSHI but decreased with an increasing FT3/FT4 ratio; the p-values for the nonlinear test were 0.62, 0.43, 0.70, 0.50, and 0.08, respectively.

**A.** The dose-response relationships between TSH and the risk of hyperuricemia; **B.** The dose-response relationships between TFQI and the risk of hyperuricemia; **C.** The dose-response relationships between TT4RI and the risk of hyperuricemia; **D.** The dose-response relationships between TSHI and the risk of hyperuricemia; **E.** The dose-response relationships between FT3/FT4 ratio and the risk of hyperuricemia. The results were adjusted for age, sex, BMI, current smoking, heavy drinking, hypertension, diabetes, dyslipidemia, ALT, AST, and eGFR.

Abbreviations: RCS, restricted cubic spline; TSH, thyroid-stimulating hormone; TFQI, thyroid feedback quantile-based index; TT4RI, thyrotrophic thyroxine resistance index; TSHI, thyroid-stimulating hormone index; FT3/FT4 ratio, free triiodothyronine to free thyroxine ratio.

**Figure S3:**
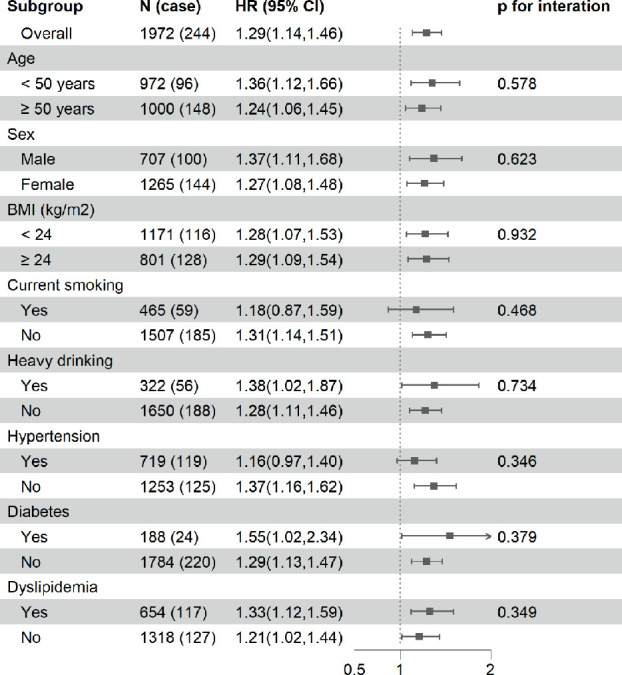
Associations of TSH and the risk of hyperuricemia in various subgroups. All the results were adjusted for age, sex, BMI, current smoking, heavy drinking, hypertension, diabetes, dyslipidemia, ALT, AST, eGFR. Abbreviations: HR, hazard ratio; CI, confidence interval; BMI, body mass index.

As shown in **Table 2**, the HR (95% CI) for hyperuricemia associated with a 1 SD higher level of TSH was 1.25 (1.11, 1.41). After adjusting for age, sex, BMI, current smoking, heavy drinking, hypertension, diabetes, dyslipidemia, ALT, AST, and eGFR, the HRs (95% CIs) for hyperuricemia were 1.26 (0.85, 1.88) in Q2, 1.62 (1.11, 2.36) in Q3, and 1.87 (1.28, 2.73) in Q4, with Q1 as the reference. As shown in **Table S4**, the association between TSH and the risk of hyperuricemia remained stable even after further adjustment for baseline eGFR and baseline SUA levels, changing the adjustment variable from BMI to waist-to-hip ratio (WHR), or using a new dataset with multiple imputation for missing data. In addition, the results of the subgroup analysis did not show any significant interaction between the subgroup variables and TSH in the development of hyperuricemia (**Figure S3**; all p-values for interaction > 0.05).

**Table 2:** Association between TSH, thyroid hormone sensitivity indices and the risk of hyperuricemia in euthyroid population

	Model 1		Model 2		Model 3	
	HR (95% CI)	p-value	HR (95% CI)	p-value	HR (95% CI)	p-value
**TSH, mIU/L**						
Q1 (<1.10)	ref.		ref.		ref.	
Q2 (≥1.10, and <1.63)	1.18 (0.79, 1.75)	0.42	1.26 (0.85, 1.88)	0.25	1.26 (0.85, 1.88)	0.26
Q3 (≥1.63, and <2.52)	1.64 (1.13, 2.37)	0.01	1.74 (1.20, 2.52)	<0.01	1.62 (1.11, 2.36)	0.01
Q4 (≥2.52)	1.68 (1.16, 2.43)	<0.01	1.86 (1.28, 2.71)	<0.01	1.87 (1.28, 2.73)	<0.01
P for trend		<0.01		<0.01		<0.01
per 1 SD	1.25 (1.11, 1.41)	<0.01	1.30 (1.15, 1.46)	<0.01	1.29 (1.14, 1.46)	<0.01
**TFQI**						
G1 (≥-1, and < -0.28)	ref.		ref.		ref.	
G2 (≥-0.28, and <0)	1.31 (0.89, 1.92)	0.17	1.31 (0.89, 1.92)	0.17	1.36 (0.93, 2.00)	0.12
G3 (≥0, and <0.28)	1.33 (0.90, 1.96)	0.15	1.37 (0.92, 2.02)	0.12	1.24 (0.83, 1.84)	0.29
G4 (≥0.28, and ≤1)	2.02 (1.41, 2.89)	<0.01	2.06 (1.44, 2.96)	<0.01	1.92 (1.33, 2.76)	<0.01
P for trend		<0.01		<0.01		<0.01
per 1 SD	1.31 (1.16, 1.49)	<0.01	1.33 (1.17, 1.51)	<0.01	1.31 (1.16, 1.49)	<0.01
**TT4RI**						
Q1 (<13.79)	ref.		ref.		ref.	
Q2 (≥13.79, and <20.51)	1.05 (0.70, 1.59)	0.81	1.08 (0.72, 1.63)	0.71	1.05 (0.69, 1.58)	0.83
Q3 (≥20.51, and <31.74)	1.64 (1.13, 2.38)	<0.01	1.76 (1.21, 2.55)	<0.01	1.67 (1.15, 2.43)	<0.01
Q4 (≥31.74)	1.89 (1.32, 2.72)	<0.01	2.04 (1.41, 2.94)	<0.01	1.93 (1.34, 2.80)	<0.01
P for trend		<0.01		<0.01		<0.01
per 1 SD	1.31 (1.16, 1.47)	<0.01	1.34 (1.19, 1.51)	<0.01	1.32 (1.17, 1.49)	<0.01
**TSHI**						
Q1 (<1.79)	ref.		ref.		ref.	
Q2 (≥1.79, and <2.21)	1.25 (0.83, 1.87)	0.29	1.27 (0.84, 1.90)	0.26	1.27 (0.84, 1.90)	0.26
Q3 (≥2.21, and <2.64)	1.79 (1.23, 2.62)	<0.01	1.88 (1.29, 2.75)	<0.01	1.81 (1.23, 2.66)	<0.01
Q4 (≥2.64)	2.01 (1.39, 2.92)	<0.01	2.14 (1.47, 3.11)	<0.01	2.02 (1.38, 2.95)	<0.01
P for trend		<0.01		<0.01		<0.01
per 1 SD	1.34 (1.18, 1.53)	<0.01	1.37 (1.21, 1.56)	<0.01	1.34 (1.18, 1.53)	<0.01
**FT3/FT4 ratio**						
Q1 (<0.30)	ref.		ref.		ref.	
Q2 (≥0.30, and <0.34)	0.90 (0.64, 1.28)	0.56	0.90 (0.64, 1.28)	0.57	0.91 (0.64, 1.29)	0.58
Q3 (≥0.34, and <0.38)	0.72 (0.50, 1.05)	0.09	0.71 (0.49, 1.03)	0.07	0.75 (0.52, 1.09)	0.13
Q4 (≥0.38)	0.84 (0.59, 1.19)	0.32	0.80 (0.56, 1.13)	0.20	0.84 (0.59, 1.19)	0.33
P for trend		0.19		0.11		0.21
per 1 SD	0.95 (0.83, 1.08)	0.42	0.92 (0.81, 1.05)	0.24	0.93 (0.82, 1.07)	0.30

Model 1: unadjusted.

Model 2: adjusted for age, sex.

Model 3: adjusted for age, sex, BMI, current smoking, heavy drinking, hypertension, diabetes, dyslipidemia, ALT, AST, eGFR.

Abbreviations: TSH, thyroid-stimulating hormone; TFQI, thyroid feedback quantile-based index; TT4RI, thyrotrophic thyroxine resistance index; TSHI, thyroid-stimulating hormone index; HR, hazard ratio; CI, confidence interval; SD, standard deviation.

**Figure S4:**
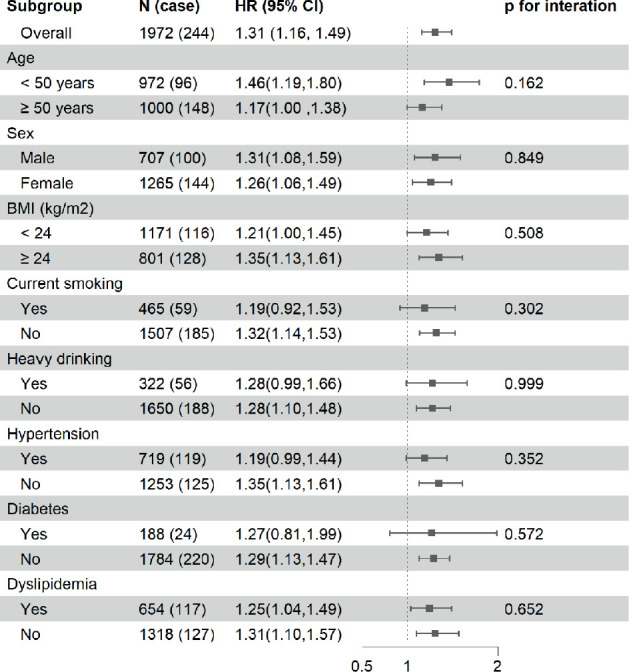
Associations of thyroid feedback quantile-based index and the risk of hyperuricemia in various subgroups. All the results were adjusted for age, sex, BMI, current smoking, heavy drinking, hypertension, diabetes, dyslipidemia, ALT, AST, eGFR. Abbreviations: HR, hazard ratio; CI, confidence interval; BMI, body mass index.

**Figure S5:**
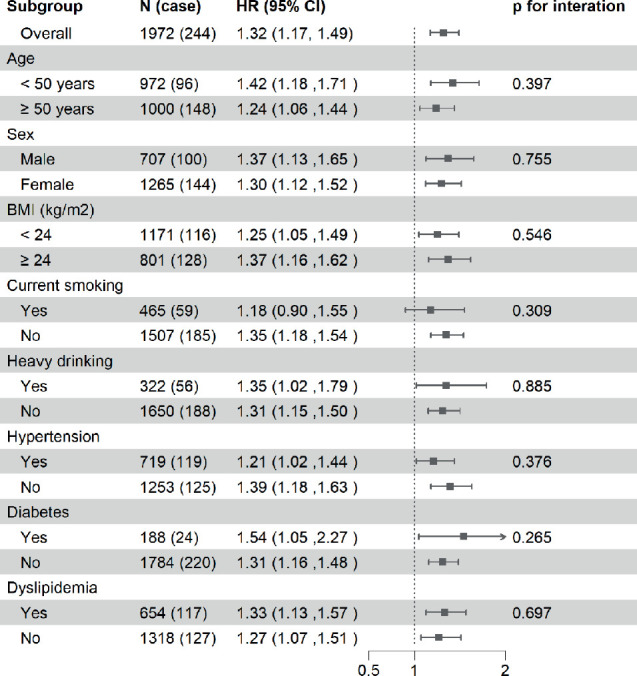
Associations of thyrotrophic thyroxine resistance index and the risk of hyperuricemia in various subgroups. All the results were adjusted for age, sex, BMI, current smoking, heavy drinking, hypertension, diabetes, dyslipidemia, ALT, AST, eGFR. Abbreviations: HR, hazard ratio; CI, confidence interval; BMI, body mass index.

**Figure S6:**
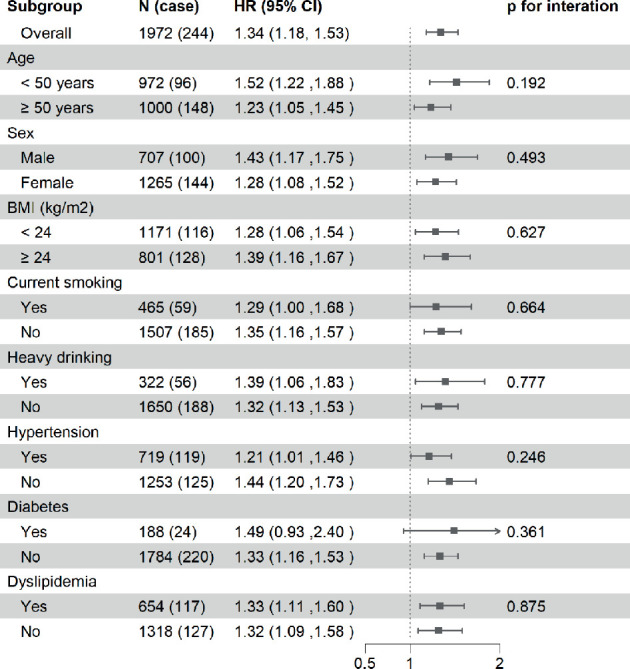
Associations of thyroid-stimulating hormone index and the risk of hyperuricemia in various subgroups. All the results were adjusted for age, sex, BMI, current smoking, heavy drinking, hypertension, diabetes, dyslipidemia, ALT, AST, eGFR. Abbreviations: HR, hazard ratio; CI, confidence interval; BMI, body mass index.

**Figure S7:**
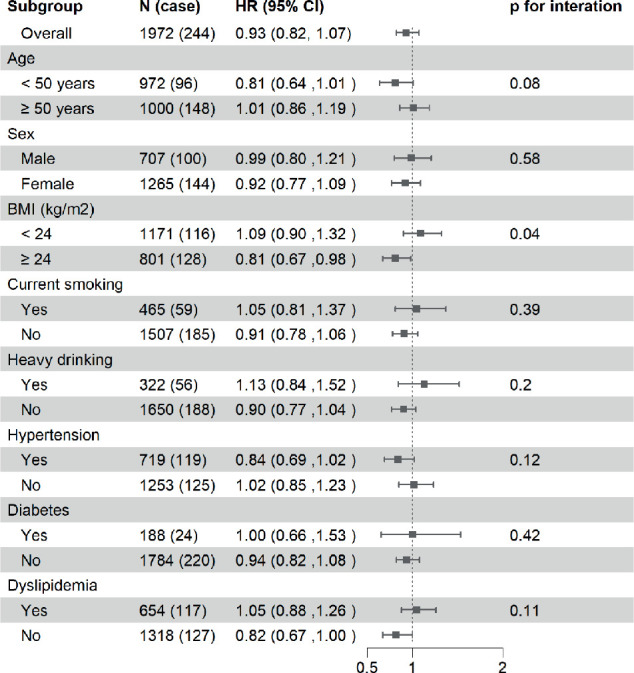
Associations of FT3/FT4 ratio and the risk of hyperuricemia in various subgroups. All the results were adjusted for age, sex, BMI, current smoking, heavy drinking, hypertension, diabetes, dyslipidemia, ALT, AST, eGFR. Abbreviations: HR, hazard ratio; CI, confidence interval; BMI, body mass index

Similarly, the HRs with 95% CIs for hyperuricemia associated with a 1 SD higher level of TFQI, TT4RI, and TSHI were 1.31 (1.16, 1.49), 1.32 (1.17, 1.49), and 1.34 (1.18, 1.53), respectively. In addition, compared with those in the lowest quartile groups, the risk of hyperuricemia was 1.92-fold (95% CI: 1.33, 2.76), 1.93-fold (95% CI: 1.34, 2.80), and 2.02-fold (95% CI: 1.38, 2.95) higher among those in the highest quartile of TFQI, TT4RI, and TSHI, respectively (**Table 2**). Further sensitivity analyses showed consistent results (**Tables S5-S7**). The subgroup analysis also did not indicate any significant interaction between the subgroup variables and TFQI, TT4RI, or TSHI on the risk of hyperuricemia (**Figure S4-S6**; all p-values for interaction > 0.05). However, FT3/FT4, which is an indirect reflection of peripheral thyroid hormone sensitivity, was not associated with the development of hyperuricemia in the present analysis (HR: 0.84; 95% CI: 0.59, 1.19). Subgroup analysis of the FT3/FT4 and hyperuricemia association is shown in **Figure S7**.

### Two-sample MR analysis

**Figure S8:**
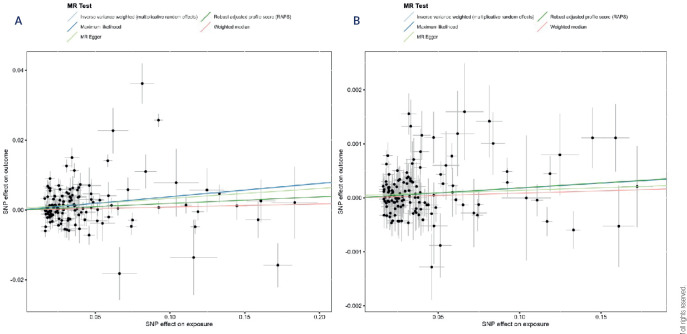
Scatter plots of MR analysis. **A.** The association of TSH and SUA; **B.** The association of TSH and gout. The slope of each line corresponds to the estimated MR effect in different models.

**Figure S9:**
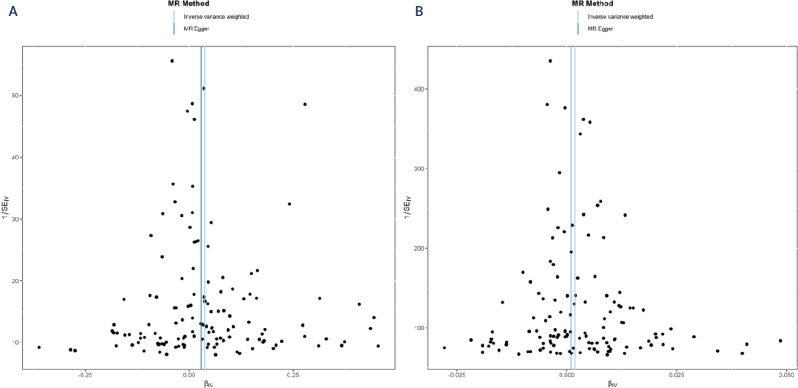
Funnel plots of MR analysis. **A.** The association of TSH and SUA; **B.** The association of TSH and gout.

**Figure S10:**
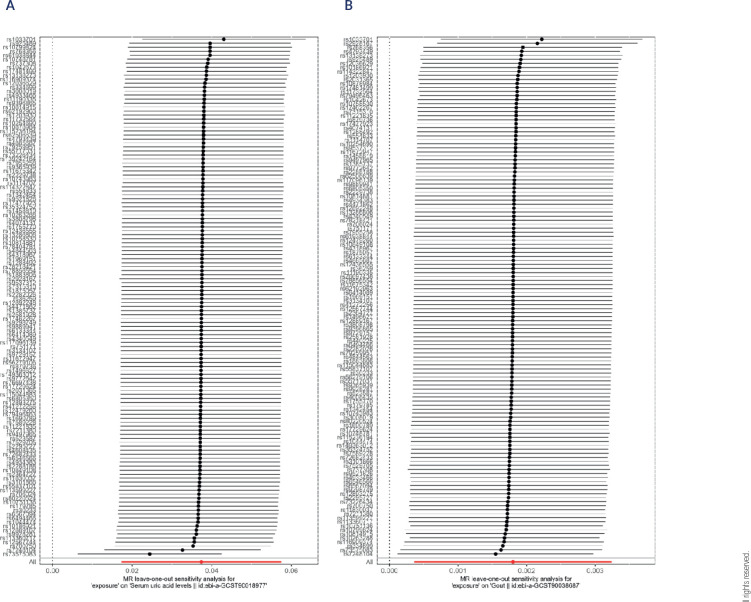
Leave-one-out plots of MR analysis. **A.** The association of TSH and SUA; **B.** The association of TSH and gout.

As shown in **Table S2**, 130 SNPs associated with TSH were included in the two-sample MR analysis. Notably, all F statistics were greater than 10, indicating a relatively low risk of weak instrument bias in the conducted MR analyses. The estimated effects and standard errors of the IVs on TSH and SUA levels or gout are presented in the scatter plots (**Figure S8**). **Table 3** shows the causal effects of TSH on SUA levels and gout. Substantial heterogeneity was detected, as indicated by Cochran’s Q test (p-value < 0.01), and the main analyses were performed using the IVW approach with the random effects model. The estimate from the IVW method indicated that genetically predicted TSH was significantly associated with SUA levels (β = 0.037; 95% CI: 0.017, 0.057) and gout (odds ratio [OR] = 1.0018; 95% CI: 1.0004, 1.0032). The MR RAPS and maximum likelihood methods confirmed the causality. The MR-PRESSO test was then performed, and the outlier-corrected results after removal of outlier SNPs were consistent with the IVW results. None of the Egger regression results were statistically significant, indicating the absence of horizontal pleiotropy in the study. The funnel plots and leave-one-out plots are shown in **Figures S9-S10**. Removal of any single SNP did not significantly change the observed association in the leave-one-out analysis.

**Table 3:** Two-sample MR analysis on the causal-effect of TSH on SUA levels and gout

Outcome	No. SNPs	Methods	β or OR (95% CI)	p-value	Q	p-value for heterogeneity	Egger intercept	p-value for pleiotropy
SUA	130	IVW (random-effect)	0.037 (0.017, 0.057)	<0.01	624.39	<0.01	0.00077	0.56
		Weighted median	0.009 (-0.008, 0.025)	0.32	-	-		
		MR Egger	0.029 (-0.007, 0.064)	0.12	622.73	<0.01		
		Maximum likelihood	0.038 (0.029, 0.047)	<0.01	-	-		
		MR RAPS	0.019 (0.002, 0.035)	0.03	-	-		
		MR-PRESSO	0.016 (0.001, 0.030)	0.03	-	-		
gout	130	IVW (random-effect)	1.0018 (1.0004, 1.0032)	0.01	185.68	<0.01	0.00006	0.44
		Weighted median	1.0018 (0.9988, 1.0029)	0.42	-	-		
		MR Egger	1.0008 (0.9984, 1.0035)	0.47	184.82	<0.01		
		Maximum likelihood	1.0018 (1.0006, 1.0030)	<0.01	-	-		
		MR RAPS	1.0018 (1.0005, 1.0032)	<0.01	-	-		
		MR-PRESSO	0.0017 (0.0003, 0.0031)	0.02	-	-		

Abbreviations: TSH, thyroid-stimulating hormone; SUA, serum uric acid; SNPs, single nucleotide polymorphisms; OR, odds ratio.

## DISCUSSION

In the present study, the results suggested that even among the euthyroid population, higher TSH levels and impaired central sensitivity to thyroid hormone were significantly associated with the risk of hyperuricemia. In addition, the results from the MR analysis provided evidence for the causal effects of TSH on SUA levels and gout. Given that the increasing prevalence of hyperuricemia has become an important disease burden worldwide, our findings are likely to have important clinical and public health implications.

The results from previous studies may partially support the current findings. It is well known that SUA levels are primarily determined by synthesis and excretion, with renal excretion of urate accounting for 60%-70% of total uric acid excretion from the body (^35^). The results from a Japanese study suggested that in the euthyroid population, TSH could increase vascular resistance at the afferent arteriole, decrease renal plasma flow, and subsequently reduce the glomerular filtration rate (^36^). In addition, Arora and cols. found that thyroid hormones could regulate renal hemodynamics, and hypothyroidism could cause reversible impairment of renal function (^37^). Furthermore, results from a clinically based study reported that thyroid hormones regulated urate metabolism by enhancing insulin sensitivity in individuals with subclinical hypothyroidism (^38^). This is because insulin increases the expression of the urate transporter urate anion transporter 1 and decreases the expression of ATP-binding cassette subfamily G member 2, resulting in increased reabsorption of urate in the body.

Several previous studies have reported that the TT4RI, TSHI, and TFQI are significantly associated with metabolic disorders, including obesity, metabolic syndrome, diabetes, and diabetes-related mortality (^12^). Results from a cross-sectional survey reported that, compared with individuals in the lowest group of thyroid hormone sensitivity indices, those in the highest group had a significantly increased prevalence of hyperuricemia (TFQI: OR = 1.18, 95% CI = 1.04-1.35; TT4RI: OR = 1.17, 95% CI = 1.08-1.27; TSHI: OR = 1.12, 95% CI = 1.04-1.21) (^39^). Additional cross-sectional studies have yielded comparable outcomes (^40^-^42^). In the present study, our findings also revealed that elevated pituitary thyroid hormone sensitivity indices (TFQI, TT4RI, and TSHI) could significantly increase the risk of hyperuricemia. However, the results from this study did not support a prospective association between the peripheral thyroid hormone sensitivity index (FT3/FT4 ratio) and the development of hyperuricemia. Recently, in a large cross-sectional study, Lu and cols. reported that each 1 SD increase in the FT3/FT4 ratio was negatively associated with hyperuricemia in euthyroid participants (males: OR = 0.11, 95% CI: 0.03-0.37; females: OR = 0.03, 95% CI = 0.01-0.21) (^42^). Given that both pituitary and peripheral thyroid hormone sensitivity are associated with the secretion of TSH, more studies are needed to further explore the prospective association between peripheral thyroid hormone sensitivity and the risk of hyperuricemia.

In recent years, two-sample MR analysis has been widely used to take SNP-exposure and SNP-outcome associations from independent GWASs and combine them into a single causal estimate (^43^). With the rapid increase in the number of GWASs investigating both TSH levels and disease outcomes, large-scale summary statistics have become widely accessible. However, the evidence for a causal relationship between serum TSH and SUA levels remains limited. Recently, Song and cols. reported a causal association between thyroid diseases (autoimmune hypothyroidism, autoimmune hyperthyroidism, thyroid nodules, and thyroid cancer) and gout using two-sample MR analysis. The results suggested that autoimmune hypothyroidism and hyperthyroidism have a causal effect on gout (IVW results: OR = 1.13, 95% CI = 1.03-1.21 for hypothyroidism; OR = 1.07, 95% CI = 1.01-1.12 for hyperthyroidism) (^44^). As expected, we also observed a causal association between TSH and SUA levels or gout in the present analysis. Therefore, the previous and present results suggest that TSH elevation is an important mechanism involved in the development of hyperuricemia.

There are several strengths in the present study, including a prospective design, long-term follow-up, and information on various covariates. Moreover, the results of two-sample MR analyses are less affected by confounders compared to traditional observational epidemiological studies, since genetic variation is stable throughout a person’s lifetime. However, several limitations should be acknowledged. First, although we have sufficiently adjusted for measured confounders, the results might still be biased due to unmeasured residual confounding (e.g., diet or medications like diuretics may alter SUA levels). However, given the relatively homogeneous dietary habits among the residents of Soochow, the potential impact of dietary factors may be limited. Second, in this study, we opted to use ECLIA to measure thyroid hormones. While LC-MS/MS is considered the gold standard in clinical chemistry, ECLIA has been shown to produce results that are highly comparable to those of LC-MS/MS. For example, analysis by Kunisue and cols. reported significant correlations for T3 (r = 0.876) and T4 (r = 0.852) measurements when comparing ECLIA with LC-MS/MS (^45^). Therefore, ECLIA has been widely used for measuring thyroid hormones in both clinical and epidemiological studies, as it offers a reliable alternative (^46^,^47^). Third, we selected a higher cutoff value for defining alcohol consumption in this analysis. One reason for this choice is that over 70% of the study participants have a customary consumption of yellow rice wine, a common practice in our population. In addition, several previous studies have shown that moderate alcohol intake is not associated with the risk of hyperuricemia or gout (^48^-^50^). For example, Li and cols. reported that moderate alcohol consumption, defined as >30 g/day for males and >15 g/day for females, did not increase the risk of hyperuricemia in Chinese adults (males: OR = 1.23, 95% CI = 0.95-1.60; females: OR = 0.90, 95% CI = 0.12-6.86) (^50^). However, the potential effects of lower levels of alcohol consumption on the association between thyroid function and hyperuricemia/gout should be further investigated in the future. Fourth, the cohort study is not a nationally representative sample, and all participants are 35-60 years of age, which limits the interpretation of results in younger, older, and other ethnic populations. Fifth, the estimates from other MR approaches (weighted median, MR Egger) were statistically insignificant, which can be ascribed to lower statistical power, indicating weak evidence for the causal relationship. In addition, the MR analyses were restricted to individuals of European ancestry, as GWAS databases for individuals of East Asian ancestry were not available.

In conclusion, the present study indicated that, even among individuals with normal thyroid function, TSH elevation and impaired central sensitivity to thyroid hormones were significantly associated with the risk of hyperuricemia. Moreover, the two-sample MR analysis provided additional evidence for the causal effects of TSH on SUA levels and gout. These findings may provide novel insight into identifying individuals at high risk of hyperuricemia and gout.

## Data Availability

the original contributions presented in this study are included in the article/Supplementary material, further inquiries can be directed to the corresponding authors.

## SUPPLEMENTARY MATERIAL

**Table S1:** The description of data sources contributing to MR study

Phenotype	PMID	GWAS ID on OpenGwas website	Consortium or Cohorts	Sample size	Year	Source
TSH	38291025	NA	the ThyroidOmics consortium	271,040	2024	https://transfer.sysepi.medizin.uni-greifswald.de/thyroidomics/datasets/
SUA	34594039	ebi-a-GCST90018977	UK Biobank	343,836	2021	https://gwas.mrcieu.ac.uk/datasets/ebi-a-GCST90018977/
Gout	33959723	ebi-a-GCST90038687	UK Biobank	484,598	2021	https://gwas.mrcieu.ac.uk/datasets/ebi-a-GCST90038687/

Abbreviations: TSH, thyroid-stimulating hormone; SUA, serum uric acid.

**Table S2:** The information for the SNPs selected in the MR analysis is significantly associated with TSH

Exposure	SNP	Effect allele	Other allele	EAF	β	SE	p-value	F statistics
TSH	rs10186921	T	C	0.556	0.0381	0.0029	1.05×10^-38^	172.60
TSH	rs10254690	T	C	0.5554	0.0168	0.0029	8.05×10^-09^	33.56
TSH	rs10263288	A	G	0.8919	-0.032	0.0046	5.37×10^-12^	48.39
TSH	rs1033701	A	G	0.2729	-0.1168	0.0032	1.00×10^-200^	1332.24
TSH	rs1042673	A	G	0.5406	-0.043	0.0029	9.41×10^-50^	219.86
TSH	rs1044474	A	G	0.5499	0.0256	0.0029	9.71×10^-19^	77.93
TSH	rs10743983	A	G	0.5451	-0.018	0.0029	7.23×10^-10^	38.53
TSH	rs10748781	A	C	0.5743	-0.059	0.003	1.97×10^-87^	386.77
TSH	rs10751136	A	G	0.5022	0.0191	0.0029	3.89×10^-11^	43.38
TSH	rs10758530	A	G	0.0547	0.0513	0.0069	1.34×10^-13^	55.28
TSH	rs10799824	A	G	0.1561	-0.1187	0.004	5.12×10^-194^	880.60
TSH	rs10814881	A	C	0.4427	-0.0288	0.0029	2.17×10^-23^	98.62
TSH	rs10814915	T	C	0.4385	0.0516	0.0029	8.80×10^-70^	316.59
TSH	rs10849108	A	G	0.1772	-0.0279	0.0038	4.25×10^-13^	53.91
TSH	rs10878984	T	C	0.3497	-0.0213	0.003	2.08×10^-12^	50.41
TSH	rs1114707	T	C	0.3372	0.0207	0.0031	3.03×10^-11^	44.59
TSH	rs11190335	A	G	0.4156	0.0166	0.003	2.79×10^-08^	30.62
TSH	rs11221835	T	C	0.2335	-0.0221	0.0034	1.33×10^-10^	42.25
TSH	rs113360717	T	C	0.0341	0.0814	0.0081	1.38×10^-23^	100.99
TSH	rs113599227	T	C	0.0444	-0.0837	0.0074	9.31×10^-30^	127.93
TSH	rs114322847	T	C	0.025	-0.1608	0.0097	2.84×10^-61^	274.81
TSH	rs115044883	A	G	0.0773	-0.0331	0.0058	1.19×10^-08^	32.57
TSH	rs115236194	A	G	0.9782	-0.0662	0.0117	1.48×10^-08^	32.01
TSH	rs11672947	T	C	0.392	-0.0236	0.0031	1.36×10^-14^	57.96
TSH	rs11675342	T	C	0.4191	0.0281	0.0029	1.34×10^-21^	93.89
TSH	rs116909374	T	C	0.0362	-0.1589	0.0085	1.11×10^-78^	349.47
TSH	rs117096139	T	C	0.0342	-0.0474	0.0084	1.47×10^-08^	31.84
TSH	rs11732564	A	G	0.2797	-0.0254	0.0033	6.50×10^-15^	59.24
TSH	rs11830037	A	C	0.0765	0.04	0.0055	3.42×10^-13^	52.89
TSH	rs12036629	A	G	0.5306	-0.0317	0.0029	1.25×10^-27^	119.49
TSH	rs1203930	A	G	0.2193	-0.0652	0.0035	1.08×10^-78^	347.02
TSH	rs12436555	A	G	0.163	-0.0216	0.0039	3.54×10^-08^	30.67
TSH	rs12479260	T	C	0.0863	-0.0385	0.0052	1.04×10^-13^	54.82
TSH	rs12567744	A	G	0.6838	0.036	0.0031	1.15×10^-30^	134.86
TSH	rs1288492	T	C	0.464	0.0185	0.0029	2.94×10^-10^	40.70
TSH	rs12889167	T	C	0.5545	0.039	0.0029	1.20×10^-40^	180.85
TSH	rs12892248	A	G	0.1249	0.0254	0.0044	7.48×10^-09^	33.32
TSH	rs12893275	T	C	0.0904	-0.0392	0.0051	1.32×10^-14^	59.08
TSH	rs13138273	A	G	0.7994	0.1107	0.0037	1.00×10^-200^	895.14
TSH	rs13286806	A	G	0.4163	-0.0181	0.003	1.42×10^-09^	36.40
TSH	rs1342454	A	G	0.0341	0.0548	0.0083	4.36×10^-11^	43.59
TSH	rs1385737	T	C	0.1254	-0.036	0.0047	2.47×10^-14^	58.67
TSH	rs139242164	T	G	0.0106	0.1158	0.0158	2.55×10^-13^	53.72
TSH	rs1458819	T	C	0.8904	0.0276	0.0048	6.34×10^-09^	33.06
TSH	rs149363012	T	C	0.0211	0.1247	0.0117	1.90×10^-26^	113.59
TSH	rs1496627	A	C	0.5794	0.0212	0.0029	5.31×10^-13^	53.44
TSH	rs1690789	T	C	0.4765	-0.0288	0.0029	5.11×10^-23^	98.62
TSH	rs17462267	A	C	0.7288	-0.0365	0.0033	6.08×10^-29^	122.34
TSH	rs17477923	T	C	0.7483	0.0609	0.0033	7.59×10^-75^	340.57
TSH	rs17481499	T	C	0.1696	0.0459	0.0038	9.13×10^-33^	145.90
TSH	rs1755770	T	C	0.6013	-0.0246	0.003	1.73×10^-16^	67.24
TSH	rs17729624	A	G	0.0998	0.0341	0.0048	1.72×10^-12^	50.47
TSH	rs179785	A	G	0.4835	0.0182	0.0029	4.35×10^-10^	39.39
TSH	rs1875057	A	G	0.3731	0.0172	0.003	8.74×10^-09^	32.87
TSH	rs1883806	T	G	0.3918	0.0198	0.0032	5.21×10^-10^	38.28
TSH	rs1969151	T	C	0.2131	0.0202	0.0035	1.24×10^-08^	33.31
TSH	rs2031365	T	C	0.2809	0.0486	0.0032	5.37×10^-52^	230.66
TSH	rs2229738	T	C	0.0946	-0.0394	0.0055	8.41×10^-13^	51.32
TSH	rs2282026	A	G	0.5295	-0.0166	0.0029	8.05×10^-09^	32.77
TSH	rs2288188	T	G	0.962	0.0719	0.0079	6.16×10^-20^	82.83
TSH	rs2295727	A	G	0.934	-0.0332	0.0059	1.51×10^-08^	31.66
TSH	rs2364727	T	C	0.1141	0.0265	0.0047	1.30×10^-08^	31.79
TSH	rs2581928	A	G	0.2653	-0.0222	0.0033	8.69×10^-12^	45.26
TSH	rs2928167	A	G	0.8648	0.133	0.0043	1.00×10^-200^	956.67
TSH	rs3008019	T	G	0.2482	-0.0309	0.0033	2.53×10^-20^	87.68
TSH	rs30233	A	G	0.5663	-0.0321	0.003	1.53×10^-27^	114.49
TSH	rs3101866	A	G	0.4778	-0.0336	0.0029	2.24×10^-31^	134.24
TSH	rs3134107	A	G	0.1786	-0.0231	0.0038	1.11×10^-09^	36.95
TSH	rs334699	A	G	0.056	-0.1447	0.0069	6.89×10^-98^	439.78
TSH	rs35324752	T	G	0.3598	-0.0198	0.0031	1.29×10^-10^	40.79
TSH	rs36259	A	G	0.7565	-0.0247	0.0035	1.86×10^-12^	49.80
TSH	rs3808798	T	G	0.2935	-0.0194	0.0032	8.36×10^-10^	36.75
TSH	rs4074131	A	G	0.8207	-0.0365	0.004	3.74×10^-20^	83.26
TSH	rs41272256	A	C	0.9213	0.0588	0.0054	8.58×10^-28^	118.57
TSH	rs4318967	T	C	0.5936	0.0191	0.0031	3.84×10^-10^	37.96
TSH	rs4340549	T	C	0.7634	-0.0233	0.0035	1.45×10^-11^	44.32
TSH	rs4471862	A	G	0.3238	-0.0248	0.0031	2.40×10^-15^	64.00
TSH	rs4608435	T	G	0.2627	-0.0231	0.0033	3.63×10^-12^	49.00
TSH	rs4793439	T	C	0.4692	0.0407	0.0029	3.57×10^-45^	196.97
TSH	rs4844563	A	C	0.6657	-0.0169	0.0031	3.54×10^-08^	29.72
TSH	rs4885687	T	G	0.7344	0.0258	0.0033	5.96×10^-15^	61.12
TSH	rs4933466	A	G	0.6025	0.033	0.003	2.67×10^-28^	121.00
TSH	rs4934383	T	C	0.6616	-0.0228	0.0031	8.64×10^-14^	54.09
TSH	rs523587	T	C	0.4296	0.0163	0.0029	2.29×10^-08^	31.59
TSH	rs554833	T	C	0.3491	0.0418	0.003	8.54×10^-44^	194.14
TSH	rs55717031	T	G	0.3057	-0.0199	0.0032	4.94×10^-10^	38.67
TSH	rs55837101	T	C	0.2249	0.0199	0.0035	1.75×10^-08^	32.33
TSH	rs56279106	A	G	0.2726	-0.0195	0.0032	2.03×10^-09^	37.13
TSH	rs6133344	T	C	0.5121	-0.024	0.0029	1.43×10^-16^	68.49
TSH	rs61938844	A	G	0.0284	0.1721	0.0097	5.52×10^-71^	314.79
TSH	rs62192963	A	G	0.1487	0.0324	0.0042	6.44×10^-15^	59.51
TSH	rs62506639	T	C	0.8097	-0.0271	0.0037	1.47×10^-13^	53.65
TSH	rs6414089	A	G	0.3578	0.0175	0.003	6.44×10^-09^	34.03
TSH	rs6494466	A	G	0.263	-0.0198	0.0033	2.08×10^-09^	36.00
TSH	rs6546566	T	C	0.6852	0.0182	0.0031	6.65×10^-09^	34.47
TSH	rs6567094	A	G	0.5543	0.0309	0.0029	2.22×10^-26^	113.53
TSH	rs6805350	T	G	0.9414	-0.0347	0.0062	2.95×10^-08^	31.32
TSH	rs700750	A	C	0.6244	0.0308	0.003	3.15×10^-25^	105.40
TSH	rs706024	A	G	0.6129	-0.0198	0.003	3.16×10^-11^	43.56
TSH	rs7211380	A	G	0.9292	0.0472	0.0056	5.20×10^-17^	71.04
TSH	rs7248104	A	G	0.4062	-0.0584	0.003	3.20×10^-87^	378.95
TSH	rs72682433	T	C	0.8965	-0.035	0.0048	2.32×10^-13^	53.17
TSH	rs7315310	A	G	0.4034	-0.0203	0.003	1.25×10^-11^	45.79
TSH	rs73226434	T	C	0.1738	-0.039	0.0038	1.23×10^-24^	105.33
TSH	rs73575083	A	G	0.6747	0.0923	0.0031	3.03×10^-199^	886.50
TSH	rs737308	T	G	0.2759	-0.0925	0.0033	1.02×10^-177^	785.69
TSH	rs751171	T	C	0.6647	-0.0358	0.0031	9.06×10^-31^	133.36
TSH	rs7529705	A	G	0.3744	0.053	0.003	9.08×10^-71^	312.11
TSH	rs7589228	A	C	0.6294	0.0264	0.003	2.43×10^-18^	77.44
TSH	rs76697438	A	G	0.0129	-0.1041	0.0151	4.75×10^-12^	47.53
TSH	rs768356	T	C	0.7996	-0.0741	0.0036	1.35×10^-92^	423.67
TSH	rs78218421	T	C	0.0627	0.044	0.0062	9.05×10^-13^	50.36
TSH	rs78404781	A	G	0.9865	0.1834	0.0155	2.86×10^-32^	140.00
TSH	rs78856554	A	G	0.0916	0.0377	0.0051	9.04×10^-14^	54.64
TSH	rs79259951	A	C	0.9314	0.0367	0.0057	1.39×10^-10^	41.46
TSH	rs79496463	T	C	0.043	0.046	0.0078	3.01×10^-09^	34.78
TSH	rs7955258	A	G	0.4162	-0.0473	0.003	1.97×10^-54^	248.59
TSH	rs80220524	T	C	0.0243	0.0619	0.0102	1.17×10^-09^	36.83
TSH	rs879736	T	C	0.1678	0.0259	0.0039	4.24×10^-11^	44.10
TSH	rs925489	T	C	0.6528	0.0749	0.003	4.50×10^-138^	623.33
TSH	rs9298749	A	C	0.6132	-0.0317	0.003	3.50×10^-26^	111.65
TSH	rs9365939	A	G	0.4561	-0.0195	0.0029	1.99×10^-11^	45.21
TSH	rs9396865	T	C	0.8409	-0.0297	0.0039	4.57×10^-14^	57.99
TSH	rs9497965	T	C	0.3956	0.0328	0.0029	9.03×10^-29^	127.92
TSH	rs9521826	A	G	0.7318	0.025	0.0033	1.88×10^-14^	57.39
TSH	rs9537312	T	C	0.2063	-0.0235	0.0036	4.37×10^-11^	42.61
TSH	rs9729157	T	C	0.6356	0.0222	0.003	1.76×10^-13^	54.76
TSH	rs9772642	T	C	0.7023	-0.0285	0.0032	1.84×10^-19^	79.32
TSH	rs9889941	A	C	0.7036	0.0184	0.0032	7.30×10^-09^	33.06
TSH	rs9928281	A	G	0.8914	-0.0344	0.005	7.94×10^-12^	47.33

Abbreviations: TSH, thyroid-stimulating hormone.

**Table S3:** The correlations of baseline TSH and thyroid hormone sensitivity indices on eGFR and SUA levels using the linear regression model

	eGFR		SUA	
	β (95% CI)	p-value	β (95% CI)	p-value
TSH	-0.539 (-0.933, -0.145)	0.01	4.236 (1.804, 6.668)	<0.01
TFQI	-1.243 (-2.203, -0.283)	<0.01	6.711 (0.788, 12.633)	0.03
TT4RI	-0.045 (-0.075, -0.015)	<0.01	0.341 (0.154, 0.527)	<0.01
TSHI	-1.008 (-1.675, -0.341)	<0.01	7.593 (3.492, 11.694)	<0.01
FT3/FT4 ratio	1.480 (-5.113, 8.072)	0.66	-75.62 (-115.14, -36.10)	<0.01

Adjusted for age, sex.

Abbreviations: TSH, thyroid-stimulating hormone; TFQI, thyroid feedback quantile-based index; TT4RI, thyrotrophic thyroxine resistance index; TSHI, thyroid-stimulating hormone index; eGFR, estimated glomerular filtration rate; SUA, serum uric acid.

**Table S4:** Sensitivity analysis of the association between thyroid-stimulating hormone index and hyperuricemia

TSHI	Model 1		Model 2		Model 3	
	HR (95% CI)	p-value	HR (95% CI)	p-value	HR (95% CI)	p-value
Consider eGFR as time dependent covariate^a^						
Q1 (<1.79)	ref.		ref.		ref.	
Q2 (≥1.79, and <2.21)	1.25 (0.83, 1.87)	0.29	1.27 (0.84, 1.90)	0.26	1.22 (0.81, 1.83)	0.34
Q3 (≥2.21, and <2.64)	1.79 (1.23, 2.62)	<0.01	1.88 (1.29, 2.75)	<0.01	1.72 (1.17, 2.52)	<0.01
Q4 (≥2.64)	2.01 (1.39, 2.92)	<0.01	2.14 (1.47, 3.11)	<0.01	1.86 (1.27, 2.71)	<0.01
by a new data-set with multiple imputation method for missing data analysis						
Q1 (<1.81)	ref.		ref.		ref.	
Q2 (≥1.81, and <2.21)	1.13 (0.77, 1.67)	0.53	1.15 (0.78, 1.69)	0.50	1.13 (0.77, 1.67)	0.54
Q3 (≥2.21, and <2.65)	1.62 (1.13, 2.32)	<0.01	1.70 (1.18, 2.44)	<0.01	1.61 (1.12, 2.32)	0.01
Q4 (≥2.65)	1.84 (1.29, 2.62)	<0.01	1.94 (1.36, 2.78)	<0.01	1.82 (1.27, 2.61)	<0.01
Additional adjustment for baseline SUA						
Q1 (<1.79)	ref.		ref.		ref.	
Q2 (≥1.79, and <2.21)	1.25 (0.83, 1.87)	0.29	1.27 (0.84, 1.90)	0.26	1.25 (0.83, 1.89)	0.29
Q3 (≥2.21, and <2.64)	1.79 (1.23, 2.62)	<0.01	1.88 (1.29, 2.75)	<0.01	1.78 (1.21, 2.63)	<0.01
Q4 (≥2.64)	2.01 (1.39, 2.92)	<0.01	2.14 (1.47, 3.11)	<0.01	2.02 (1.38, 2.95)	<0.01
The adjustment variable BMI was replaced by WHR						
Q1 (<1.79)	ref.		ref.		ref.	
Q2 (≥1.79, and <2.21)	1.25 (0.83, 1.87)	0.29	1.27 (0.84, 1.90)	0.26	1.28 (0.85, 1.92)	0.24
Q3 (≥2.21, and <2.64)	1.79 (1.23, 2.62)	<0.01	1.88 (1.29, 2.75)	<0.01	1.83 (1.25, 2.68)	<0.01
Q4 (≥2.64)	2.01 (1.39, 2.92)	<0.01	2.14 (1.47, 3.11)	<0.01	2.03 (1.39, 2.96)	<0.01

Model 1: unadjusted.

Model 2: adjusted for age, sex.

Model 3: adjusted for age, sex, BMI(WHR), current smoking, heavy drinking, hypertension, diabetes, dyslipidemia, ALT, AST, baseline eGFR (time dependent covariate).

a Using Time Dependent Covariates in the Cox Model.

Abbreviations: TSHI, thyroid-stimulating hormone index; HR, hazard ratio; CI, confidence interval; eGFR, estimated glomerular filtration rate; HUA, hyperuricemia; BMI, body mass index; WHR, waist-to-hip ratio

**Table S5:** Sensitivity analysis of the association between thyroid feedback quantile-based index and hyperuricemia

TFQI	Model 1		Model 2		Model 3	
	HR (95% CI)	p-value	HR (95% CI)	p-value	HR (95% CI)	p-value
Consider eGFR as time dependent covariate^a^						
G1 (≥ -1, and <-0.28)	ref.		ref.		ref.	
G2 (≥-0.28, and <0)	1.31 (0.89, 1.92)	0.17	1.31 (0.89, 1.92)	0.17	1.35 (0.92, 1.98)	0.13
G3 (≥0, and <0.28)	1.33 (0.90, 1.96)	0.15	1.37 (0.92, 2.02)	0.12	1.13 (0.77, 1.68)	0.53
G4 (≥0.28, and ≤1)	2.02 (1.41, 2.89)	<0.01	2.06 (1.44, 2.96)	<0.01	1.77 (1.23, 2.55)	<0.01
by a new data-set with multiple imputation method for missing data analysis						
G1 (≥ -1, and <-0.28)	ref.		ref.		ref.	
G2 (≥-0.28, and <0)	1.38 (0.95, 1.99)	0.09	1.39 (0.96, 2.00)	0.08	1.44 (0.997, 2.09)	0.05
G3 (≥0, and <0.28)	1.26 (0.86, 1.85)	0.23	1.30 (0.89, 1.91)	0.18	1.15 (0.78, 1.70)	0.47
G4 (≥0.28, and ≤1)	1.96 (1.38, 2.78)	<0.01	1.99 (1.40, 2.83)	<0.01	1.86 (1.31, 2.66)	<0.01
Additional adjustment for baseline SUA						
G1 (≥ -1, and <-0.28)	ref.		ref.		ref.	
G2 (≥-0.28, and <0)	1.31 (0.89, 1.92)	0.17	1.31 (0.89, 1.92)	0.17	1.34 (0.91, 1.98)	0.14
G3 (≥0, and <0.28)	1.33 (0.90, 1.96)	0.15	1.37 (0.92, 2.02)	0.12	1.17 (0.79, 1.75)	0.43
G4 (≥0.28, and ≤1)	2.02 (1.41, 2.89)	<0.01	2.06 (1.44, 2.96)	<0.01	1.91 (1.33, 2.76)	<0.01
The adjustment variable BMI was replaced by WHR						
G1 (≥-1, and <-0.28)	ref.		ref.		ref.	
G2 (≥-0.28, and <0)	1.31 (0.89, 1.92)	0.17	1.31 (0.89, 1.92)	0.17	1.37 (0.93, 2.01)	0.11
G3 (≥0, and <0.28)	1.33 (0.90, 1.96)	0.15	1.37 (0.92, 2.02)	0.12	1.24 (0.84, 1.84)	0.28
G4 (≥0.28, and ≤1)	2.02 (1.41, 2.89)	<0.01	2.06 (1.44, 2.96)	<0.01	1.90 (1.32, 2.74)	<0.01

Model 1: unadjusted.

Model 2: adjusted for age, sex.

Model 3: adjusted for age, sex, BMI(WHR), current smoking, heavy drinking, hypertension, diabetes, dyslipidemia, ALT, AST, baseline eGFR (time dependent covariate).

a Using Time Dependent Covariates in the Cox Model.

Abbreviations: TFQI, thyroid feedback quantile-based index; HR, hazard ratio; CI, confidence interval; eGFR, estimated glomerular filtration rate; HUA, hyperuricemia; BMI, body mass index; WHR, waist-to-hip ratio.

**Table S6:** Sensitivity analysis of the association between thyrotrophic thyroxine resistance index and hyperuricemia

TT4RI	Model 1		Model 2		Model 3	
	HR (95% CI)	p-value	HR (95% CI)	p-value	HR (95% CI)	p-value
Consider eGFR as time dependent covariate^a^						
Q1 (<13.79)	ref.		ref.		ref.	
Q2 (≥13.79, and <20.51)	1.05 (0.70, 1.59)	0.81	1.08 (0.72, 1.63)	0.71	1.01 (0.67, 1.52)	0.97
Q3 (≥20.51, and <31.74)	1.64 (1.13, 2.38)	<0.01	1.76 (1.21, 2.55)	<0.01	1.59 (1.09, 2.31)	0.02
Q4 (≥31.74)	1.89 (1.32, 2.72)	<0.01	2.04 (1.41, 2.94)	<0.01	1.82 (1.26, 2.63)	<0.01
by a new data-set with multiple imputation method for missing data analysis						
Q1 (<13.93)	ref.		ref.		ref.	
Q2 (≥13.93, and <20.59)	1.01 (0.68, 1.50)	0.98	1.03 (0.70, 1.54)	0.87	1.00 (0.67, 1.49)	0.99
Q3 (≥20.59, and <31.89)	1.56 (1.09, 2.24)	0.02	1.66 (1.16, 2.39)	<0.01	1.57 (1.09, 2.26)	0.02
Q4 (≥31.89)	1.83 (1.29, 2.60)	<0.01	1.97 (1.38, 2.80)	<0.01	1.86 (1.30, 2.66)	<0.01
Additional adjustment for baseline SUA						
Q1 (<13.79)	ref.		ref.		ref.	
Q2 (≥13.79, and <20.51)	1.05 (0.70, 1.59)	0.81	1.08 (0.72, 1.63)	0.71	1.03 (0.68, 1.56)	0.88
Q3 (≥20.51, and <31.74)	1.64 (1.13, 2.38)	<0.01	1.76 (1.21, 2.55)	<0.01	1.62 (1.11, 2.36)	0.01
Q4 (≥31.74)	1.89 (1.32, 2.72)	<0.01	2.04 (1.41, 2.94)	<0.01	1.86 (1.28, 2.70)	<0.01
The adjustment variable BMI was replaced by WHR						
Q1 (<13.79)	ref.		ref.		ref.	
Q2 (≥13.79, and <20.51)	1.05 (0.70, 1.59)	0.81	1.08 (0.72, 1.63)	0.71	1.04 (0.69, 1.57)	0.84
Q3 (≥20.51, and <31.74)	1.64 (1.13, 2.38)	<0.01	1.76 (1.21, 2.55)	<0.01	1.66 (1.14, 2.41)	<0.01
Q4 (≥31.74)	1.89 (1.32, 2.72)	<0.01	2.04 (1.41, 2.94)	<0.01	1.97 (1.36, 2.85)	<0.01

Model 1: unadjusted.

Model 2: adjusted for age, sex.

Model 3: adjusted for age, sex, BMI (WHR), current smoking, heavy drinking, hypertension, diabetes, dyslipidemia, ALT, AST, baseline eGFR (time dependent covariate).

a Using Time Dependent Covariates in the Cox Model.

Abbreviations: TT4RI, thyrotrophic thyroxine resistance index; HR, hazard ratio; CI, confidence interval; eGFR, estimated glomerular filtration rate; HUA, hyperuricemia; BMI, body mass index; WHR, waist-to-hip ratio.

**Table S7:** Sensitivity analysis of the association between thyroid-stimulating hormone index and hyperuricemia

TSHI	Model 1		Model 2		Model 3	
	HR (95% CI)	p-value	HR (95% CI)	p-value	HR (95% CI)	p-value
Consider eGFR as time dependent covariate^a^						
Q1 (<1.79)	ref.		ref.		ref.	
Q2 (≥1.79, and <2.21)	1.25 (0.83, 1.87)	0.29	1.27 (0.84, 1.90)	0.26	1.22 (0.81, 1.83)	0.34
Q3 (≥2.21, and <2.64)	1.79 (1.23, 2.62)	<0.01	1.88 (1.29, 2.75)	<0.01	1.72 (1.17, 2.52)	<0.01
Q4 (≥2.64)	2.01 (1.39, 2.92)	<0.01	2.14 (1.47, 3.11)	<0.01	1.86 (1.27, 2.71)	<0.01
by a new data-set with multiple imputation method for missing data analysis						
Q1 (<1.81)	ref.		ref.		ref.	
Q2 (≥1.81, and <2.21)	1.13 (0.77, 1.67)	0.53	1.15 (0.78, 1.69)	0.50	1.13 (0.77, 1.67)	0.54
Q3 (≥2.21, and <2.65)	1.62 (1.13, 2.32)	<0.01	1.70 (1.18, 2.44)	<0.01	1.61 (1.12, 2.32)	0.01
Q4 (≥2.65)	1.84 (1.29, 2.62)	<0.01	1.94 (1.36, 2.78)	<0.01	1.82 (1.27, 2.61)	<0.01
Additional adjustment for baseline SUA						
Q1 (<1.79)	ref.		ref.		ref.	
Q2 (≥1.79, and <2.21)	1.25 (0.83, 1.87)	0.29	1.27 (0.84, 1.90)	0.26	1.25 (0.83, 1.89)	0.29
Q3 (≥2.21, and <2.64)	1.79 (1.23, 2.62)	<0.01	1.88 (1.29, 2.75)	<0.01	1.78 (1.21, 2.63)	<0.01
Q4 (≥2.64)	2.01 (1.39, 2.92)	<0.01	2.14 (1.47, 3.11)	<0.01	2.02 (1.38, 2.95)	<0.01
The adjustment variable BMI was replaced by WHR						
Q1 (<1.79)	ref.		ref.		ref.	
Q2 (≥1.79, and <2.21)	1.25 (0.83, 1.87)	0.29	1.27 (0.84, 1.90)	0.26	1.28 (0.85, 1.92)	0.24
Q3 (≥2.21, and <2.64)	1.79 (1.23, 2.62)	<0.01	1.88 (1.29, 2.75)	<0.01	1.83 (1.25, 2.68)	<0.01
Q4 (≥2.64)	2.01 (1.39, 2.92)	<0.01	2.14 (1.47, 3.11)	<0.01	2.03 (1.39, 2.96)	<0.01

Model 1: unadjusted.

Model 2: adjusted for age, sex.

Model 3: adjusted for age, sex, BMI(WHR), current smoking, heavy drinking, hypertension, diabetes, dyslipidemia, ALT, AST, baseline eGFR (time dependent covariate).

a Using Time Dependent Covariates in the Cox Model.

Abbreviations: TSHI, thyroid-stimulating hormone index; HR, hazard ratio; CI, confidence interval; eGFR, estimated glomerular filtration rate; HUA, hyperuricemia; BMI, body mass index; WHR, waist-to-hip ratio.

## Data Availability Statement:

the original contributions presented in this study are included in the article/Supplementary material, further inquiries can be directed to the corresponding authors.
